# Effectiveness and Implementation of Obstetric Triage During Pregnancy and Childbirth: A Systematic Review

**DOI:** 10.7759/cureus.89215

**Published:** 2025-08-01

**Authors:** Nihal Eltayeb Abdalla Elsheikh, Hanady ME M Osman, Sahar Altayeb Alfaki Ahmed, Shahinaz Abdelgaium Elsamani Mohamed, Ryan Osman Alhessen Saidahmed, Rewan Samy Said Awad Eltalkhawy, Hadeel Mohammed Ahmed Elbashir

**Affiliations:** 1 Obstetrics and Gynecology, Najran Armed Forces Hospital, Ministry of Defense Health Services, Najran, SAU; 2 Quality and Patient Safety, Najran Armed Forces Hospital, Ministry of Defense Health Services, Najran, SAU; 3 Obstetrics and Gynecology, King Khaled Majmaah Hospital, Riyadh, SAU; 4 Obstetrics and Gynaecology, Royal Surrey County Hospital, Surrey, GBR; 5 Obstetrics and Gynaecology, Nottingham University Hospitals, Nottingham, GBR; 6 Obstetrics and Gynecology, Al Kharj Armed Forces Hospital, Al-Kharj, SAU

**Keywords:** implementation science, maternal healthcare, obstetric triage, pregnancy emergencies, quality improvement, triage protocols

## Abstract

Obstetric triage systems play a vital role in ensuring timely care for pregnant women, yet their implementation and effectiveness vary across healthcare settings. This systematic review synthesizes evidence on the impact of standardized obstetric triage systems on care timeliness, staff competency, and maternal-fetal outcomes, while examining barriers and facilitators to successful implementation in diverse contexts.

Following Preferred Reporting Items for Systematic Reviews and Meta-Analyses (PRISMA) guidelines, we conducted a systematic search across six databases (PubMed, Embase, Scopus, Web of Science, CINAHL, IEEE Xplore). Eligible studies evaluated obstetric triage interventions, reported quantitative or mixed-methods data on implementation/effectiveness outcomes (e.g., time to assessment, length of stay (LOS), staff knowledge), and were peer-reviewed. 11 studies met inclusion criteria and were evaluated using the Risk of Bias in Non-randomized Studies of Interventions (ROBINS-I) tool for risk of bias; seven were rated as low risk. Due to heterogeneity in interventions, outcomes, and settings (high- vs low-resource), a narrative synthesis was performed. Key findings demonstrated that standardized triage tools significantly reduced the time from patient arrival to initial evaluation by a healthcare provider (time to provider assessment) and LOS in high-resource settings. In low-resource contexts, locally adapted models reduced median waiting times from 40 minutes to five minutes, though systemic barriers like understaffing persisted. Successful implementation relied on staff training and workflow integration, while variability in adoption reflected organizational culture. Limitations included heterogeneity precluding meta-analysis and under-representation of low-resource settings. Obstetric triage standardization enhances care efficiency, but effectiveness depends on contextual adaptation and multidisciplinary engagement. Future research should prioritize randomized controlled trials (RCTs), cost-effectiveness analyses, and tailored strategies for low-resource settings.

## Introduction and background

Timely and well-prioritized care during pregnancy and childbirth is essential for achieving the best outcomes for both mothers and newborns [1,2]. In real-world clinical settings, pregnant women often arrive with a range of concerns, from mild discomfort to urgent, life-threatening complications, requiring quick and accurate assessment [3].

Obstetric triage provides a structured method to evaluate and prioritize these patients. Unlike general emergency triage, which is designed for all patient types, obstetric triage uses pregnancy-specific tools such as the Maternal-Fetal Triage Index (MFTI) and the Obstetric Triage Acuity Scale (OTAS) [4]. The MFTI is a five-level system that helps categorize pregnant women based on urgency, while the OTAS assigns acuity levels according to specific obstetric symptoms and signs. Both aim to quickly identify conditions like pre-eclampsia, severe bleeding, and preterm labor, where every minute can be critical for maternal and fetal survival [5]. For example, a woman arriving with severe abdominal pain and elevated blood pressure can be rapidly triaged and treated before complications escalate, potentially saving both her life and her baby’s.

Research and hospital audits suggest that using structured triage systems can reduce waiting times, improve staff decision-making, and enhance patient outcomes [4,6]. However, results vary across settings, and many studies report limited impact on more severe outcomes like ICU admissions or perinatal mortality. Additionally, most available evidence comes from high-resource hospitals. In contrast, facilities in low- and middle-income countries (LMICs) often face significant challenges-such as limited staffing, inadequate training, and lack of standardized protocols-that make consistent triage implementation difficult [7].

This systematic review aims to synthesize current evidence on both the effectiveness and practical implementation of obstetric triage systems. We assess how different tools affect outcomes, such as waiting time, triage accuracy, and maternal-fetal health, while also examining barriers and enablers in various healthcare contexts. By bringing this evidence together, we hope to inform clinical practice, support policy decisions, and identify areas for future research to improve emergency obstetric care across diverse settings.

## Review

Methodology

This systematic review was conducted in accordance with the Preferred Reporting Items for Systematic Reviews and Meta-Analyses (PRISMA) guidelines to evaluate the effectiveness and implementation of obstetric triage systems during pregnancy and childbirth [8]. The methodology was designed to ensure comprehensive literature coverage, rigorous study selection, and transparent reporting while accounting for clinical and methodological heterogeneity across studies.

Eligibility Criteria

Studies were included if they evaluated obstetric triage systems in pregnancy or childbirth settings, reported quantitative or mixed-methods data on implementation or effectiveness outcomes, and were published in peer-reviewed journals. Eligible outcomes included time-sensitive metrics (e.g., time to assessment, length of stay (LOS)), staff knowledge, and system adoption rates. All study designs were considered, including randomized controlled trials (RCTs), quasi-experimental studies, and quality improvement (QI) projects. Exclusion criteria encompassed studies focusing solely on general emergency triage without obstetric applications, qualitative-only designs, and non-peer-reviewed publications such as editorials or conference abstracts.

Search Strategy and Databases

A systematic search was executed across six databases (PubMed, Embase, Scopus, Web of Science, CINAHL, and IEEE Xplore). The search strategy combined Medical Subject Headings (MeSH) and free-text terms, including "obstetric triage," "maternal triage," and "pregnancy emergency assessment," adapted for each database. To minimize retrieval bias, backward and forward citation tracking of included studies was performed, and gray literature sources were screened. The detailed search strategy is attached in Appendix 1.

Study Selection Process

The selection process followed PRISMA guidelines. After duplicate removal, two independent reviewers screened titles/abstracts against eligibility criteria. Full texts of potentially relevant studies were retrieved and assessed independently. Discrepancies were resolved through discussion or third-reviewer consultation. The process was documented using a PRISMA flowchart, detailing the number of records identified, excluded, and included at each stage.

Data Extraction and Management

For data extraction and management, a standardized form was developed to systematically capture key elements from each included study, including study characteristics such as author, year, country, and design; population details including sample size and setting; intervention components such as the specific triage tool used and implementation strategy; reported outcomes encompassing both primary and secondary endpoints; and key findings. To ensure accuracy and minimize bias, two independent reviewers performed the data extractions, with any discrepancies resolved through discussion and consensus. In cases where data were missing or unclear, attempts were made to contact the original study authors to obtain the necessary information, thereby enhancing the completeness and reliability of the extracted data. This rigorous approach to data extraction and management helped maintain consistency and transparency throughout the review process.

Quality Assessment

The Risk of Bias in Non-randomized Studies of Interventions (ROBINS-I) tool was applied to assess methodological quality across seven domains: confounding, selection bias, intervention classification, deviations from intended interventions, missing data, outcome measurement, and selective reporting [9]. Studies were categorized as low, moderate, serious, or critical risk of bias.

Data Synthesis Approach

A narrative synthesis approach was adopted for this systematic review due to substantial heterogeneity observed across three key dimensions: the interventions examined (which included varied triage tools such as MFTI, OTAS, and Birmingham Symptom-Specific Obstetric Triage System (BSOTS)), the outcomes measured (encompassing diverse metrics ranging from time-based indicators to staff knowledge and adoption rates), and the study settings (spanning both high-income and low-resource hospital environments). This significant clinical and methodological variability made meta-analysis inappropriate, as statistical pooling of such heterogeneous data would produce results lacking clinical interpretability and validity. Instead, the review employed a thematic synthesis strategy, systematically organizing and analyzing findings to identify overarching patterns related to implementation facilitators and barriers, as well as trends in intervention effectiveness across different contexts. This approach allowed for a more nuanced understanding of how obstetric triage systems function in real-world settings while respecting the inherent diversity of the evidence base.

Ethical Considerations

As only published data were synthesized, no ethical approval was required. Proper attribution and transparent reporting were maintained throughout.

Results

Study Selection Process

**Figure 1:**
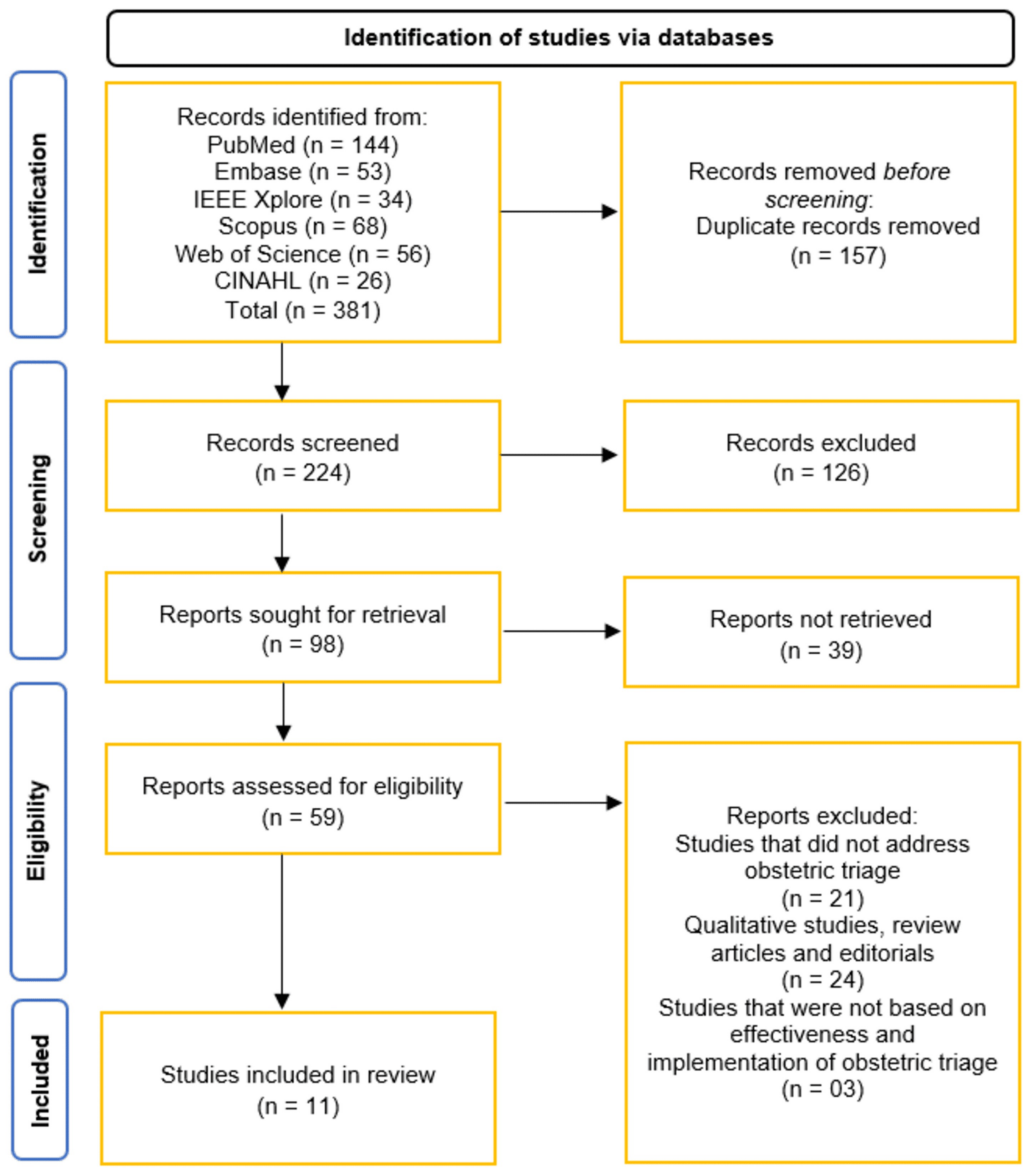
Overview of study selection – from initial records to final included studies

The systematic review initially identified 381 records from six databases (PubMed, Embase, IEEE Xplore, Scopus, Web of Science, and CINAHL), with 157 duplicates removed prior to screening. After screening 224 records, 126 were excluded, leaving 98 reports for retrieval. Of these, 39 were not retrieved, and an additional 48 were excluded for not addressing obstetric triage (n = 21), being qualitative studies, reviews, or editorials (n = 24), or lacking focus on effectiveness and implementation (n = 3). Ultimately, 59 full-text reports were assessed for eligibility, with 11 studies meeting the inclusion criteria and incorporated into the review (Figure 1) [10-20].

Characteristics of Included Studies

The review included 11 studies evaluating the effectiveness and implementation of obstetric triage systems across diverse settings, including high-income countries (USA, Canada, UK, Australia) and low-resource settings (Uganda, Ghana) [10-20]. The study designs varied, encompassing prospective observational studies, QI projects, mixed-methods evaluations, and pre-post intervention studies [10-15,18,20]. Sample sizes ranged from 26 midwives in an education evaluation to over 12,000 triage visits [12,16]. The populations primarily consisted of pregnant women presenting with obstetric emergencies or complications, with a focus on severe hypertension, pre-eclampsia, and general obstetric complaints.

The triage interventions included standardized tools such as the MFTI, the OTAS, and symptom-specific systems like the BSOTS [10-13,16]. Comparators were predominantly pre-implementation data or historical cohorts. Key outcome measures included timeliness of care (e.g., time to provider assessment, LOS), staff knowledge, and system adoption rates. A detailed summary of study characteristics is provided in Table 1.

**Table 1:** Details of included studies – from sample characteristics to outcomes of obstetric triage interventions ED: Emergency department; MAU: Maternity assessment unit; OTAS: Obstetric Triage Acuity Scale; MFTI: Maternal-Fetal Triage Index; OTDA: Obstetric Triage Decision Aid; BSOTS: Birmingham Symptom-Specific Obstetric Triage System; QI: Quality improvement; LOS: Length of stay; NoMAD: Normalization MeAsure Development; ACOG: American College of Obstetricians and Gynecologists; ICC: Intraclass correlation coefficient; IQR: Interquartile range

Study	Country	Study Design	Setting	Sample Size	Population Characteristics	Type of Triage Intervention	Comparator (if any)	Outcome Measures	Main Findings
Hoffmann et al. [10]	USA	Prospective observational study	Large urban inner-city academic hospital	370 patients (pre intervention, 2019) and 254 patients (post intervention, 2021)	Pregnant women with severe pre-eclampsia diagnosed by severe hypertension	Implementation of the MFTI with standardized education for staff	Pre-implementation outcomes (2019)	Time to provider assessment; time to magnesium sulfate prophylaxis; time to antihypertensive administration	Implementation of MFTI significantly reduced time to provider assessment (median 44 minutes to 17 minutes); time to magnesium sulfate administration (161 minutes to 127 minutes); and time to antihypertensive administration (101 minutes to 66 minutes). MFTI improved timeliness of assessment and treatment for severe hypertension.
Quaile [11]	USA (southeastern region)	QI project with pre and post testing	Two hospitals within a large multi-campus hospital system	Obstetric triage nurses who participated in pre/post tests	Obstetric triage nurses with > 2 years of obstetrics experience	MFTI implementation with educational module	Pre-implementation scores and triage times	Nursing knowledge (pre vs post test scores); timeliness of care (triage times pre vs post implementation)	Nursing knowledge improved from mean 79% to 95%; timeliness of care improved from mean 19 minutes pre implementation to 10.4 minutes post implementation
Smithson et al. [12]	Canada	Prospective before-and-after study	Obstetric triage unit	Pre implementation: 8,085 visits; Post OTAS: 8,131 visits; Post fast-track: 12,576 visits total	Pregnant women presenting to obstetric triage	Implementation of standardized 5-category OTAS and fast-track for OTAS 4 and 5 (lower-acuity) patients	Pre-implementation period (usual care without OTAS or fast-track)	Median LOS before and after OTAS and fast-track implementation	OTAS implementation reduced median LOS from 105 to 101 minutes (P = 0.04); fast-track further reduced LOS for lower-acuity patients to 73 minutes (P = 0.005) and overall LOS to 98 minutes (6.9% reduction; P < 0.001)
Kodama et al. [13]	USA	Retrospective QI study	Urban, tertiary labor and delivery triage unit	1,305 (historical cohort), 1,374 (study cohort)	Pregnant women ≥16 weeks’ gestation presenting to triage from Dec 2017-Feb 2018 and Dec 2018-Feb 2019	MFTI 5-level triage system implemented with formal nurse education	Historical cohort before MFTI implementation	Primary: Duration of triage evaluation (presentation to completion of history and physical; Secondary: Admission rates	Implementation of MFTI resulted in slightly longer overall triage evaluations (64 vs 61 minutes, P = 0.02); higher priority patients had shorter evaluation times; admission rates were highest for priority 1 (89.3%) and priority 5 (92.6%) groups; supports ACOG recommendation for triage tools
Kenyon et al. [14]	UK	Mixed-methods evaluation (structured audit, inter-operator reliability study, focus group, questionnaires, national survey)	Large UK maternity unit	Structured audit: 994 notes before and after implementation; Inter-operator reliability study; Focus group (n = 12); Questionnaire (n = 79 midwives, 53 responded); National survey (135 units, 85 responded)	Pregnant women attending the maternity unit; midwives working in triage	Newly designed obstetric triage system including standard clinical assessment by midwife within 15 minutes and assignment to a 4-category clinical urgency scale with training program	Pre-implementation data (before intervention)	% of women seen within 15 minutes of attendance; time to subsequent care; inter-operator reliability; midwives’ views; national survey of practice	Increase in % of women seen within 15 minutes from 39% to 54% (RR 1.4, P < 0.0001); excellent inter-operator reliability (ICC 0.961); midwives reported improved management and safety; national survey showed majority (81%) of units did not use a clinical assessment-based triage system
McCarthy et al. [15]	Australia	Pre-post implementation study	ED and MAU in a hospital	2,829 unscheduled maternity presentations (ED: 708, MAU: 2,121)	Pregnant and postpartum women presenting unscheduled to ED and MAU; staff included midwives and emergency nurses	OTDA, a decision aid tool with targeted questions generating triage scores for 10 common pregnancy complaints	Pre-implementation data (staff survey and waiting times)	Adoption rate of OTDA, staff confidence and competence; waiting times; prioritization of care	OTDA was used in 88.1% of cases (MAU: 93.2%, ED: 72.7%; P < 0.001 MAU saw significant improvement in women within minutes to staff confidence and competence improved reduced under-triage risk ED prioritization MAU.
Vasilevski et al. [16]	Australia	Multi-method study (pre-implementation education evaluations, post-implementation clinical data audit, staff focus groups)	Tertiary maternity service (Maternity Assessment Centre)	Education evaluations: 26 midwives; Clinical audit: 660 women; Focus groups: 9 staff	Midwives working in the Maternity Assessment Centre and women attending during implementation	BSOTS	Pre-implementation audits (baseline)	Time to triage; time to care; staff perceptions	BSOTS improved time to triage and time to care outcomes; staff valued its standardization, especially new midwives; lack of knowledge among staff (especially medical staff) was a barrier
Forshaw et al. [17]	Uganda	Prospective audit	Mulago National Referral Hospital, obstetric admissions department	Not specified	Pregnant women presenting for obstetric care	‘Traffic Light System’ triage tool	None	Time spent in admissions; identification of urgent cases; use of triage system	The ‘Traffic Light System’ was not used at the first checkpoint; informal triage occurred 46% of the time; vital signs were often not recorded; the system may not be suitable for early triage stages.
Goodman et al. [18]	Ghana	Pre-post intervention study (with multiple time point comparisons)	Referral hospital in Accra	Not stated	Pregnant women presenting for delivery or obstetric care	Obstetric triage improvement program including training, QI monitoring, and establishment of a dedicated triage pavilion	Baseline data before intervention (2012)	Waiting time from arrival to assessment; documentation of care plan	Median waiting time reduced from 40 minutes to 5 minutes (P < 0.001); care plan documentation improved from 51% to 96%; delays associated with admission and disease acuity were eliminated
Goodman et al. [19]	Ghana	Prospective cohort study	Ridge Regional Hospital, Accra (largest referral hospital in Ghana Health System)	1,082 women	Pregnant women presenting with complications over a 10-week period; referred from 108 facilities	Existing obstetric triage process (arrival-to-evaluation times analyzed)	Observational analysis of subgroups, e.g., by reason for referral, time of arrival)	Wait times based on reason for referral; time and day of arrival; concurrent triage area volume	Median wait time was 40 minutes (IQR 15–100); only 22% evaluated within 10 minutes; time-sensitive conditions (hypertension, hemorrhage, second-stage labor) were prioritized but still not within 10 minutes standard; longer waits associated with night shift, latent labor, non-time-sensitive referrals.
Ramaswamy et al. [20]	Ghana	Retrospective mixed-methods evaluation	Tema General Hospital, Greater Accra (high-volume maternity hospital)	Not stated	Pregnant women presenting for obstetric assessment	Theory-based implementation of obstetric triage blending implementation science and QI concepts	Baseline data (pre implementation)	Timeliness of first assessment (arrival-to-assessment waiting time); accuracy of diagnosis; appropriateness of care plan; embeddedness (NoMAD assessment of normalization process theory constructs)	Median arrival-to-assessment waiting time decreased from 70.5 minutes (IQR 30.0–443.0) at baseline to 6.0 minutes post implementation and 5.0 minutes at sustainment; accuracy of diagnosis improved from baseline to 77.9% at sustainment; appropriateness of care plan improved from 66.0% to 78.9%; staff perceived triage to be well integrated and sustainable

Effectiveness of Obstetric Triage Interventions

The implementation of standardized triage systems demonstrated significant improvements in timeliness of care. Hoffmann et al. reported a reduction in median time to provider assessment from 44 minutes to 17 minutes (P < 0.001) and time to antihypertensive administration from 101 minutes to 66 minutes (P < 0.001) following MFTI implementation [10]. Similarly, Quaile observed a decrease in mean triage time from 19 minutes to 10.4 minutes post intervention, alongside improved nursing knowledge (79% to 95%) [11]. Smithson et al. found that OTAS implementation reduced median LOS from 105 minutes to 101 minutes (p = 0.04), with further reductions to 73 minutes for lower-acuity patients using a fast-track system (p = 0.005) [12].

In contrast, Kodama et al. reported a slight increase in overall triage evaluation duration (64 vs 61 minutes, P = 0.02), though higher-priority patients were evaluated faster (Priority 1: 57 minutes), supporting effective acuity stratification [13]. Kenyon et al. noted a 15% absolute increase in women seen within 15 minutes (39% to 54%, P < 0.0001), with high inter-operator reliability (intraclass correlation coefficient (ICC) = 0.961) [14]. McCarthy et al. achieved an 88.1% adoption rate for their Obstetric Triage Decision Aid (OTDA), with 78% of women in maternity units seen within 15 minutes post intervention (P < 0.001) [15].

In low-resource settings, Goodman et al. reduced median waiting times from 40 minutes to five minutes (P < 0.001) through a triage improvement program, while Ramaswamy et al. sustained a reduction from 70.5 minutes to five minutes post implementation [18,20]. However, Forshaw et al. reported ineffective use of the Traffic Light System in Uganda, with informal triage persisting in 46% of cases [17]. A synthesis of intervention outcomes is presented in Table 2.

**Table 2:** Summary of intervention outcomes MFTI: Maternal-Fetal Triage Index; OTAS: Obstetric Triage Acuity Scale; OTDA: Obstetric Triage Decision Aid; BSOTS: Birmingham Symptom-Specific Obstetric Triage System; LOS: Length of stay; NoMAD: Normalization MeAsure Development; IQR: Interquartile range; ICC: Intraclass correlation coefficient; MAU: Maternity assessment unit; ED: Emergency department; QI: Quality improvement

Study	Type of Triage Intervention	Outcome(s) Measured	Effectiveness Outcome	Implementation Findings	Statistical Significance (p-value/CI)
Hoffmann et al. [10]	MFTI	Time to provider assessment; time to magnesium sulfate administration; time to antihypertensive medication administration	Median time to provider assessment decreased from 44 minutes to 17 minutes; median time to magnesium sulfate administration decreased from 161 minutes to 127 minutes; median time to antihypertensive medication administration decreased from 101 minutes to 66 minutes	Implementation of MFTI with standardized education improved timeliness of assessment and treatment for severe hypertension in pregnancy	Time to provider assessment: P < 0.001; time to magnesium sulfate administration: P = 0.001; time to antihypertensive administration: P < 0.001
Quaile [11]	MFTI with educational module	Nursing knowledge of triage assessment; timeliness of care (triage time)	Nursing knowledge increased from 79% (pre-test mean) to 95% (post-test mean); timeliness of care improved from a mean triage time of 19 minutes (pre implementation) to 10.4 minutes (post implementation)	Implementation included an educational module followed by MFTI use; nurses completed pre test and post test, and triage times were compared pre and post implementation	Not reported
Smithson et al. [12]	Standardized 5-category OTAS and fast-track for lower-acuity patients (OTAS 4 and 5)	LOS	Median LOS decreased from 105 minutes (IQR 52-178) to 101 minutes (IQR 49-175) after OTAS implementation; further decreased to 73 minutes (IQR 40-140) for lower-acuity patients with fast-track; overall LOS reduced to 98 minutes (IQR 47-172), a 6.9% reduction	OTAS implemented prospectively; computerized simulation modeling used to implement fast-track for OTAS 4 and 5 patients	P = 0.04 (OTAS implementation); P = .005 (lower-acuity fast-track); P < 0.001 (overall LOS)
Kodama et al. [13]	MFTI (5-level triage system)	Duration of triage evaluation (time from presentation to completion of history and physical); rates of labor and delivery admissions	Overall duration of triage evaluation increased in study cohort compared to historical cohort (64 minutes vs 61 minutes); within study cohort, higher priority patients had shorter evaluation times (Priority 1: 57 minutes; Priority 2: 66 minutes; Priority 3: 63 minutes; Priority 4: 62 minutes; Priority 5: 83 minutes); admission rates higher in Priority 1 (89.3%) and Priority 5 (92.6%)	Implementation included formal education for nurses and assignment of priority levels at triage presentation; demonstrated effective prioritization for higher acuity patients despite overall increased evaluation duration.	Overall duration difference: P = 0.02; evaluation time by priority level: P < 0.001; admission rates by priority: P < 0.001
Kenyon et al. [14]	Standardized obstetric triage system with 4-category urgency scale and structured midwife assessment within 15 minutes of attendance	% of women seen within 15 minutes; time to subsequent care; inter-operator reliability; midwives’ perceived safety and department management; national prevalence of similar triage systems	Increase in women seen within 15 minutes: from 39% to 54%; excellent inter-operator reliability (ICC = 0.961)	Midwives reported improved safety and departmental management; training was well-received; national survey revealed lack of standardized triage in majority of UK units (81%)	P < 0.0001 for primary outcome; ICC = 0.961 (95% CI: 0.91–0.99)- RR = 1.4 (95% CI: 1.2–1.7)
McCarthy et al. [15]	OTDA	Adoption rate; staff confidence; staff competence; waiting times (women seen within 15 minutes)	Adoption rate: 88.1% overall (MAU: 93.2%, ED: 72.7%); increased percentage of women seen within 15 minutes in MAU (from 42.0% to 78.0%); improved staff confidence and competence in triage	Different implementation approaches required across ED and MAU; high adoption, more in MAU; reduced clinical risk from under-triage in ED; improved prioritization of care in MAU	P < 0.001 (MAU vs ED adoption rate); P = 0.002 (staff confidence); P = 0.004 (staff competence)
Vasilevski et al. [16]	BSOTS	Time to triage; time to care; staff perceptions	Improved time to triage and care; outcomes mostly adhered to auditable standards	Staff valued the standardized approach, especially helpful for new midwives; lack of awareness among medical staff was a barrier to effective implementation	Not reported
Forshaw et al. [17]	Traffic Light System	Time spent in admissions; identification of urgent cases; recording of vital signs	Ineffective in current use – not utilized; many vital signs not recorded	Not used at first checkpoint; informal triage used 46% of the time; system focus not suitable for early patient journey stage	Not reported
Goodman et al. [18]	Obstetric triage improvement program with triage pavilion	Waiting time from arrival to assessment; documentation of care plan; time of admission delays	Median waiting time reduced from 40 minutes (IQR 15–100) to 5 minutes (IQR 2–6); care plan documentation increased from 51% to 96%; delays eliminated	Implemented over 5 years using Active Implementation Framework; included training, QI tools, dedicated triage pavilion with midwives; locally led and sustained	P < 0.001 for waiting time reduction
Goodman et al. [19]	Obstetric triage at arrival to referral hospital (evaluation upon arrival based on reason for referral, time/day of arrival, and concurrent triage volume)	Arrival-to-evaluation (wait) time; proportion of women evaluated within 10 minutes; factors associated with delay	Median wait time was 40 minutes (IQR 15–100); only 22% evaluated within 10 minutes; time-sensitive cases (e.g. hypertensive disorders, hemorrhage, second-stage labor) were seen faster than baseline	Longer wait times at night shift, for latent labor, and non-time-sensitive risks; all groups failed to meet the international standard of evaluation within 10 minutes	Not reported
Ramaswamy et al. [20]	Theory-based obstetric triage implementation using concepts from implementation science and QI	Waiting time (arrival-to-assessment); accuracy of diagnosis; appropriateness of care plan; embeddedness (per NoMAD survey)	Median waiting time decreased from 70.5 minutes (IQR 30.0–443.0) to 6.0 minutes post implementation and 5.0 minutes at sustainment; diagnosis accuracy improved from 75.7% to 77.9%; appropriateness of care plans improved from 66.0% to 78.9%	Intervention successfully integrated into clinical workflow; high staff acceptance and perceived normalization per NoMAD; sustained improvements observed 12 months post implementation	Not reported

Implementation Findings and Barriers

Successful implementation was associated with structured education, staff training, and integration into clinical workflows. Hoffmann et al. and Quaile emphasized the role of standardized education for staff in improving triage accuracy and efficiency [10,11]. Kenyon et al. highlighted midwives' positive perceptions of safety and departmental management post intervention, though a national survey revealed 81% of UK units lacked standardized systems [14].

Barriers included variability in adoption across settings. McCarthy et al. noted higher OTDA uptake in maternity units (93.2%) compared to emergency departments (EDs) (72.7%, P < 0.001), attributed to differing workflows [15]. Vasilevski et al. identified lack of awareness among medical staff as a barrier to BSOTS implementation, despite midwives valuing its standardization [16]. In Ghana, Goodman et al. and Ramaswamy et al. demonstrated that locally led, theory-based interventions with dedicated triage spaces were critical for sustainment [18,20].

Summary of Key Outcomes

Overall, obstetric triage interventions consistently improved timeliness of care and staff competency, particularly in high-resource settings. Reductions in time to assessment and LOS were statistically significant across multiple studies [10,12,14]. In low-resource contexts, interventions requiring minimal infrastructure (e.g., triage pavilions, staff training) showed promise, though contextual challenges like informal triage and resource limitations persisted [17-20].

Quality Assessment Results

The risk of bias assessment using the ROBINS-I tool revealed that most studies (7/11) were rated as low risk, demonstrating robust methodologies with minimal confounding, selection bias, and deviations from interventions [10,12-15,18,20]. Three studies had a moderate risk, primarily due to confounding or moderate selection bias, while Forshaw et al. was the sole study with serious risk, attributed to significant biases in selection, deviations, and outcome measurement [11,16,17,19]. Notably, no study exhibited critical bias, and all maintained low risk in intervention classification and selective reporting, supporting the reliability of their findings despite contextual limitations (Table 3).

**Table 3:** Quality assessment of included studies using ROBINS-I tool ROBINS-I: Risk of Bias in Non-randomized Studies of Interventions

Study	Confounding	Selection Bias	Intervention Classification	Deviations from Interventions	Missing Data	Outcome Measurement	Selective Reporting	Overall Risk of Bias
Hoffmann et al. [10]	Low	Low	Low	Low	Low	Low	Low	Low
Quaile [11]	Serious	Moderate	Low	Moderate	Low	Moderate	Low	Moderate
Smithson et al. [12]	Low	Low	Low	Low	Low	Low	Low	Low
Kodama et al. [13]	Low	Low	Low	Low	Low	Low	Low	Low
Kenyon et al. [14]	Low	Low	Low	Low	Low	Low	Low	Low
McCarthy et al. [15]	Low	Low	Low	Low	Low	Low	Low	Low
Vasilevski et al. [16]	Serious	Moderate	Low	Moderate	Moderate	Moderate	Low	Moderate
Forshaw et al. [17]	Serious	Serious	Low	Serious	Moderate	Serious	Low	Serious
Goodman et al. [18]	Low	Low	Low	Low	Low	Low	Low	Low
Goodman et al. [19]	Serious	Moderate	Low	Moderate	Moderate	Moderate	Low	Moderate
Ramaswamy et al. [20]	Low	Low	Low	Low	Low	Low	Low	Low

Discussion

Clinical Effectiveness of Standardized Triage Systems

The findings of this review demonstrate that standardized obstetric triage systems significantly improve the timeliness and quality of care for pregnant women across diverse healthcare settings. The evidence suggests that well-implemented triage interventions, particularly those incorporating structured education and clinical workflow integration, lead to measurable reductions in critical time-sensitive outcomes such as time to provider assessment, administration of life-saving medications, and overall LOS. Hoffmann et al. and Quaile showed that the MFTI, when paired with staff training, reduced median assessment times by over 50% and improved nursing knowledge substantially [10,11]. These results align with existing literature emphasizing the importance of standardized protocols in obstetric emergencies, where delays can have severe consequences [21]. Similarly, Smithson et al. demonstrated that the OTAS with a fast-track system for lower-acuity patients not only reduced median LOS but also optimized resource allocation, a finding consistent with studies on acuity-based triage in EDs [12,22].

Implementation Factors and Organizational Context

However, the effectiveness of these interventions varied depending on contextual factors, such as healthcare infrastructure and staff adherence. For instance, Kodama et al. reported a paradoxical increase in overall triage duration post-MFTI implementation, likely due to the tool's emphasis on comprehensive evaluations for high-risk patients [13]. This suggests that while acuity stratification improves prioritization, it may also introduce additional steps that extend process times, a trade-off noted in other complex triage systems [23]. In low-resource settings, the challenges were more pronounced. Forshaw et al. found that the Traffic Light System in Uganda was largely ineffective, with informal triage persisting due to systemic barriers like understaffing and inadequate training [17]. This echoes broader literature on implementation gaps in LMICs, where triage tools often fail without parallel investments in workforce capacity and institutional support [24]. Conversely, Goodman et al. and Ramaswamy et al. achieved remarkable success in Ghana by combining triage interventions with locally led QI initiatives, underscoring the importance of contextual adaptation, a principle well-documented in implementation science [18,20,25].

Barriers and Enablers Across Settings

The review also highlights the critical role of staff training and engagement in triage system success. Studies like Kenyon et al. and McCarthy et al. reported improved staff confidence and inter-operator reliability when triage tools were introduced alongside comprehensive training programs [14,15]. These findings corroborate evidence that clinician buy-in and competency are pivotal for the sustained use of standardized tools [26]. Yet, barriers such as resistance from medical staff, as observed by Vasilevski et al. with the BSOTS, reveal persistent challenges in multidisciplinary adoption [16]. This aligns with studies on change management in healthcare, where siloed workflows and hierarchical structures often hinder uniform implementation [27].

Implications for Practice and Policy

The variability in adoption rates across settings further underscores the influence of organizational culture. McCarthy et al. noted significantly higher uptake of the OTDA in maternity units (93.2%) compared to EDs (72.7%), reflecting the latter's competing priorities and less tailored workflows [15]. Similar disparities have been reported in hybrid triage models, where emergency care settings struggle to integrate obstetric-specific protocols [28]. On the other hand, Goodman et al. and Ramaswamy et al. demonstrated that dedicated triage spaces, like pavilions staffed by midwives, could eliminate delays and improve care plan documentation-a model supported by WHO guidelines for maternal care in LMICs [18,20,29].

The risk of bias assessment revealed that most studies (7/11) were methodologically robust, with low risk of confounding and selection bias. However, the moderate risk in studies like Quaile and Vasilevski et al. often stemmed from non-randomized designs and self-reported outcomes, common limitations in QI research [11,16,30]. Forshaw et al. stood out as the sole study with serious bias, likely due to its audit design and lack of a comparator, which limits the generalizability of its findings [17]. Despite these limitations, the consistency of positive outcomes across high- and low-resource settings strengthens the case for obstetric triage standardization.

When compared to existing literature, this review's findings reinforce the transformative potential of triage systems in maternal healthcare. The observed reductions in time to critical interventions mirror results from studies on trauma and emergency triage, where standardized protocols save lives by minimizing delays [31]. However, obstetric triage uniquely addresses the "third delay" (delays in receiving appropriate care after reaching a facility), a concept pivotal in maternal mortality frameworks [32]. The success of programs in Ghana, as reported by Goodman et al., directly tackles this delay, offering a replicable model for LMICs grappling with overcrowded referral systems [18,19]. Conversely, the mixed results in high-resource settings suggest that even well-resourced systems must balance thoroughness with speed, a challenge highlighted in obstetric rapid response literature [13,33].

Limitations

This review has several limitations. First, the predominance of pre-post and observational study designs introduces risks of confounding and temporal biases, though the ROBINS-I tool was used to assess and account for these. Second, substantial heterogeneity in outcome measures (e.g., time to assessment vs. staff knowledge) limited the feasibility of meta-analysis, thereby constraining quantitative synthesis. Third, the under-representation of studies from LMICs (only 4 out of 11) may skew findings toward high-resource settings. Fourth, potential publication bias remains a concern, as studies with positive outcomes are more likely to be published. Finally, limitations in our own review process should be acknowledged, including the restriction to English-language publications, which may have excluded relevant non-English studies, and the possibility of selection bias during screening and data extraction-despite being mitigated by independent dual-review procedures.

## Conclusions

Standardized obstetric triage systems can improve timely care, staff performance, and clinical outcomes, especially when supported by training and locally adapted protocols. While tools like MFTI and OTAS show strong results in high-resource settings, low-resource environments benefit more from simplified, context-appropriate models. However, the current evidence, largely observational, heterogeneous, and limited in LMIC representation, warrants cautious interpretation. Future research should prioritize well-designed trials, cost-effectiveness studies, and practical implementation strategies to guide broader adoption. Strengthening triage systems remains a vital step toward reducing preventable maternal and neonatal harm globally.

## Appendices

Appendix 1

**Table 4:** Search strategy

Database	Search Terms	Search Dates
PubMed	("obstetric triage" OR "maternity triage" OR "perinatal triage") AND ("pregnancy" OR "childbirth" OR "labor" OR "maternal care") AND ("implementation" OR "effectiveness")	July 01, 2025
Embase	('obstetric triage' OR 'maternity triage' OR 'perinatal triage') AND ('pregnancy'/exp OR 'childbirth'/exp OR 'labor'/exp) AND ('implementation' OR 'effectiveness')	July 01, 2025
Scopus	TITLE-ABS-KEY ("obstetric triage" OR "maternity triage" OR "perinatal triage") AND TITLE-ABS-KEY ("pregnancy" OR "childbirth" OR "labor" OR "maternal care") AND TITLE-ABS-KEY ("implementation" OR "effectiveness")	July 02, 2025
Web of Science	TS=("obstetric triage" OR "maternity triage" OR "perinatal triage") AND TS=("pregnancy" OR "childbirth" OR "labor" OR "maternal care") AND TS=("implementation" OR "effectiveness")	July 02, 2025
CINAHL	("obstetric triage" OR "maternity triage" OR "perinatal triage") AND ("pregnancy" OR "childbirth" OR "labor" OR "maternal care") AND ("implementation" OR "effectiveness")	July 02, 2025
IEEE Xplore	("obstetric triage" OR "maternity triage") AND ("pregnancy" OR "childbirth") AND ("implementation" OR "effectiveness")	July 03, 2025
